# Trends in parameterization, economics and host behaviour in influenza pandemic modelling: a review and reporting protocol

**DOI:** 10.1186/1742-7622-10-3

**Published:** 2013-05-07

**Authors:** Luis R Carrasco, Mark Jit, Mark I Chen, Vernon J Lee, George J Milne, Alex R Cook

**Affiliations:** 1 Department of Statistics and Applied Probability, National University of Singapore, Singapore, Singapore; 2 Department of Biological Sciences, National University of Singapore, Singapore, Singapore; 3 Modelling and Economics Unit, Health Protection Agency, London, UK; 4 Saw Swee Hock School of Public Health, National University of Singapore, Singapore, Singapore; 5 Program in Emerging Infectious Diseases, Duke-NUS Graduate Medical School, Singapore, Singapore; 6 Communicable Disease Centre, Tan Tock Seng Hospital, Singapore, Singapore; 7 Biodefence Centre, Ministry of Defence, Singapore, Singapore; 8 Centre for Health Services Research, National University of Singapore, Singapore, Singapore; 9 School of Computer Science and Software Engineering, The University of Western Australia, Crawley, Western Australia, Australia; 10 Program in Health Services and Systems Research, Duke-NUS Graduate Medical School, Singapore, Singapore

**Keywords:** Bayesian inference, Behaviour, Economic analysis, Epistemology of simulation, Influenza, Pandemic modelling

## Abstract

**Background:**

The volume of influenza pandemic modelling studies has increased dramatically in the last decade. Many models incorporate now sophisticated parameterization and validation techniques, economic analyses and the behaviour of individuals.

**Methods:**

We reviewed trends in these aspects in models for influenza pandemic preparedness that aimed to generate policy insights for epidemic management and were published from 2000 to September 2011, i.e. before and after the 2009 pandemic.

**Results:**

We find that many influenza pandemics models rely on parameters from previous modelling studies, models are rarely validated using observed data and are seldom applied to low-income countries. Mechanisms for international data sharing would be necessary to facilitate a wider adoption of model validation. The variety of modelling decisions makes it difficult to compare and evaluate models systematically.

**Conclusions:**

We propose a model Characteristics, Construction, Parameterization and Validation aspects protocol (CCPV protocol) to contribute to the systematisation of the reporting of models with an emphasis on the incorporation of economic aspects and host behaviour. Model reporting, as already exists in many other fields of modelling, would increase confidence in model results, and transparency in their assessment and comparison.

## Introduction

Influenza pandemics are overwhelmingly large scale phenomena that may result in high morbidity, mortality and large economic impacts worldwide. The influenza pandemic of 1918–9 is believed to have caused excess mortality of 20–40 million people [1]. Influenza pandemics have occurred during the 20th century and beginning of the 21st century at intervals of between 10 and 40 years, with the latest pandemics occurring in 1918–9, 1957–8, 1968–9 [2] and 2009–10 [3]. Pharmaceutical and public health measures can help mitigate the impacts of pandemics [4,5] and were implemented by many governments during the last pandemic in 2009–10 [6,7].

Because empirical or field studies of population-level strategies to control or mitigate influenza pandemics are generally either infeasible (e.g. controlling movement of people within a city) or unethical (e.g. withholding vaccination of subpopulations to assess the effect on transmission), modelling is one of the only suitable methodologies to enable multiple hypothetical pandemic preparedness and mitigation scenarios to be assessed. Epidemic models are especially useful to address epidemiological, economic, and individuals’ behavioural questions [8-10]. The usefulness of epidemic models in directing mitigation efforts has been supported by empirical findings that have echoed previous modelling predictions. For instance, models predicted that reduced international air travel would be unlikely to stop an influenza pandemic [11], a finding later verified empirically during the 2009 H1N1 pandemic [12,13]; other models predicted the potential of antiviral prophylaxis and contact tracing to control small outbreaks [5], a prediction also verified in real-life outbreaks in semi-closed army camps [14].

For epidemic models to produce reasonable predictions on the course of the epidemic and how it can be controlled, we need to be confident that the model captures the essential mechanisms that drive the epidemic dynamics [15]. It is therefore essential to parameterize the model from available data [15,16] and validate the model to increase its credibility [17]. One of the main focuses of this review is to evaluate the trends in the construction and validation of mechanistic models — models that explicitly incorporate the mechanisms or processes underlying the outcomes of the system — of infection dynamics for influenza pandemic preparedness, control and mitigation. Traditionally, the main approach for mechanistic modelling of influenza pandemics has been based on compartmental models (Table 1) represented by systems of differential equations. Compartmental models represented the dynamics of a host-disease system for which a tractable analytical solution could in principle be derived through mathematical methods [8,18]. It was not until the widespread availability of modern computing power that more complex compartmental models for which analytical solutions could not be derived and agent-based models (ABMs, Table 1) explored using computer simulation became an attractive alternative. Recent modelling work, dealing with the threat of an influenza pandemic of avian origin (A-H5N1), with the severe acute respiratory syndrome (SARS) crisis in 2003 and the H1N1 2009 pandemic, has exemplified the use of both models solved analytically and through simulation [3,5,19,20].

**Table 1:** Definitions of model types

Compartmental epidemic models	Models that divide the population according to states relevant to the disease studied and represent the rates at which individuals change state. These models are widely used in epidemic modelling and can be represented by systems of differential or difference equations or stochastic rates. For instance a SIR compartmental model would divide the population according to whether the individuals are susceptible (S), infectious (I) or recovered (R). Basic compartmental models assume perfect mixing between homogeneous individuals but can be expanded to account for instance for different transmission rates between ages (age-structured compartmental models), or other heterogeneities
Network or random graph models	Network (graph) models are models that characterize the relationships between individuals. Infection occurs only between individuals (nodes) that have a connection between them (arcs or edges).
Agent-based models	These models simulate the actions and interactions of autonomous agents with the aim to observe patterns of aggregation resulting from such interaction. Their relevance in epidemic modelling stems from their capacity to represent interactions and decisions at the individual level.
Metapopulation models	Metapopulation models originate from ecology and are used to represent distinct populations distributed in separated and discrete habitat patches. The populations can interact through migration. These models are useful in epidemic modelling by making the patches represent cities or other levels of spatial aggregation, thus allowing for the consideration of spatial structure. Although in their original application in ecology they did not consider the dynamics within patches, they are amenable of incorporating the epidemic dynamics within each patch, e.g. using compartmental models.
Game theoretic models	Models that study the decisions of an individual when the outcome of such decisions depends on the decisions of other individuals. These models study when cooperation or defection would arise from the interaction between individuals given certain circumstances. They can be useful in epidemic modelling to explore the incentives that humans face regarding vaccination, wearing face masks or adopting other preventative behaviour.
Optimal control and stochastic programming models	These are dynamic optimization techniques that aim to find the optimal way to control a system over time. In the case of epidemic modelling, they are useful to investigate for instance the optimal deployment of vaccines or antivirals over time to minimize the disease burden or the overall costs generated by the epidemic. These models are different to the other models that assume a level of control that is independent of the state of the system. By contrast, these models allow control to very depending of the final outcome or the state of the system.
Partial or general computable equilibrium models	Partial equilibrium models are economic models based on the equilibrium of the supply and demand of a market assuming that the prices and quantities traded in other markets do not vary. Computable equilibrium models (CGE), by contrast, consider the interactions between the markets composing an economy and study the price equilibrium in all the markets considered.

Pandemic preparedness, control and mitigation modelling has heretofore been reviewed [21-25]. These reviews show a bewildering array of models that have been introduced, especially since the 2009 pandemic, with different purposes, outcomes and structures. Despite the usefulness of modelling, few public health practitioners or decision makers undergo explicit training in modelling techniques. When combined with the rapid growth in modelling capabilities driven by increasing computing power, and the multitude of different disciplines – e.g. economics, psychology, genetics – that contribute to modelling epidemics, this makes it daunting to keep abreast of all that modelling is capable of. To facilitate model understanding, this review focuses in three pandemic modelling aspects that are recently experimenting substantial innovations: parameterization and validation, economic aspects [26] and behaviour of the hosts [27]. Given the diversity of new techniques in these aspects of modelling, a review of common traits would be very helpful for non-expert users to determine which modelling techniques are most useful to address the decisions they face. In addition, a protocol to guide the reporting of these aspects together with model construction would help modellers and policy makers compare and evaluate models. To this end, we review and classify models for influenza pandemic preparedness from January 2000 to September 2011, and use the resulting analysis to develop a simple guiding protocol for reporting modelling decisions.

## Methods

### Search strategy and selection criteria

We searched Google Scholar, PubMed and ISI Web of Knowledge to identify articles focusing on influenza pandemic modelling to inform management strategies (see Additional file 1: Figure S1 in the electronic supplementary material (ESM) for a PRISMA flow diagram [28]). Our search criterion was: contains pandemic AND model* AND influenza AND policy OR policies. Our eligibility criteria were articles that: (i) were published in peer reviewed journals from January 2000 to September 2011; (ii) aimed to advise policy makers and made policy recommendations about pandemic influenza preparedness, mitigation or control; and (iii) employed mechanistic models to derive those insights. We further excluded cost-effectiveness and decision tree studies that did not incorporate disease transmission dynamics. The search in PubMed retrieved 72 articles, ISI Web of Knowledge 128, and Google Scholar 19,200 results. After an additional query refinement in Google Scholar (adding to the previous query the terms: AND preparedness OR strateg* AND simulation OR compartment*), screening of articles and further full-text assessment for their eligibility (Additional file 1: Figure S1 in ESM), 91 articles were selected for the analysis.

### Classification and evaluation of modelling traits

We classify models into several major groups: compartmental epidemic models, network models, agent-based models, metapopulation models, game theoretic models, optimal control models and partial or general computable equilibrium models (definitions of the models can be found in Table 1). In some instances models can conform to several categories: e.g. compartmental models combined with metapopulation models.

In addition to classifying the models, we evaluated traits common to several taxa consistent with the focus of the review on evaluating the trends in parameterization and validation, incorporation of economic aspects and host behaviour (Table 2). We evaluated capacity of the models to answer (i) epidemiological questions: for instance, how many people and which age groups were expected to become infected, hospitalized and die as a result of infection during the pandemic? To what extent would control and treatment interventions mitigate this impact?; (ii) health economic questions: what would be the economic impacts of the pandemic and which interventions would represent better value for money to reduce the health and economic impacts?; (iii) behavioural questions: would changes in the behaviour of the individuals during the pandemic influence the effectiveness of the interventions?

**Table 2:** Processes for model construction and validation

Parameterization	The process of selecting the values or distributions of the model parameters based on empirical data, usually with a random component. Rigorous parameterization is fundamental since the value of the parameters largely determines the behaviour and predictions of the model.
Sensitivity and uncertainty analysis	The study of the influence of the parameter values of the models on the model outcomes. Sensitivity analysis can vary one parameter at a time (univariate) or multiple (multivariate). The comparison of the model predictions with the baseline parameter values and the modified values gives an idea of how sensitive the model is to a certain parameter. Sensitivity analysis is useful because enhances the communication of the model, tests the robustness of the results allowing the evaluation of our confidence in the predictions, increases our understanding of the system and allows detection of implementation errors. Uncertainty analysis evaluates the model response for the plausible range of the parameters. Uncertainty analysis provides information on what variable generates more uncertainty in the model and can help to direct data collection efforts.
Validation	The process of investigating whether model predictions are likely to be accurate. Two main types of validation can be distinguished: structural and predictive validation [29]. Structural validity requires that the model reproduces the observed system behaviour and is constructed in accordance with the way the real system operates, i.e. is consistent and based on theory. Predictive validation requires that the model predicts accurately data that were not used in its construction. It has also been argued that the credibility of a model might be provided by the credentials of the model building techniques, that sometimes involve contrary-to-fact principles that increase the reliability of the results [30].
Least squares	Standard data fitting procedure that consists on the minimization of the squares of the difference between the observed data points and the fitted value provided by the model.
Maximum likelihood estimation	Method to estimate the parameters of a model based on data. This method chooses values for which the probability of generating the observed data is highest, given the model.
Bayesian inference	Method of statistical inference to estimate the parameters of a model combining prior belief and the evidence observed. As more evidence is gathered the prior distribution is modified into the posterior distribution that represents the uncertainty over the parameters value.
Markov chain Monte Carlo (MCMC)	MCMC are algorithms that can be used to sample the posterior distribution for Bayesian inference and are useful because they allow to sample from multi-dimensional distributions of observations.
Particle filtering	Particle filtering is a parameterization technique based on the simulation and sequential weighting of a sample of parameter values according to their consistency with the observed data. Particle filters are normally used to parameterize Bayesian models in which variables that cannot be observed are inferred by the model through connection in a Markov chain.
Calibration	Here we define calibration as an iterative comparison between model predictions and observed data (e.g. attack rates, R_0_) without the use of standard statistical inference methods. After comparison, simulation of the model for different parameter values is performed and compared with the former predictions to see if an improvement in their agreement is obtained.

To classify models by their construction and validation techniques, we evaluated whether they (i) incorporated an assessment of the sensitivity of their results to model parameters and assumptions, (ii) were parameterized using parameters directly from other models, and (iii) were validated from empirical data. To that end, the articles were categorized according to the type of model used (Table 1), population heterogeneity level considered, parameterization procedure, consideration of economic impacts, inclusion of human behaviour and performance of validation or sensitivity analysis (see the ESM for a full list of the models and their characteristics).

### Standard reporting protocol

To facilitate the systematization and comparison of models we develop a guiding protocol for reporting model general Characteristics, Construction, Parameterization and Validation aspects (CCPV protocol), derived from the results of the review. The protocol builds upon previous protocols to describe ABMs [31] and retains the description of technical aspects of the models that facilitate its understanding and reproduction, for instance describing the aim of the model, scale, structure, model type, dynamic aspects, initialization, data inputs, the way individuals and their interactions are considered or the inclusion of stochasticity (Table 3). The protocol was then extensively expanded to include aspects relative to models construction, parameterization, sensitivity analysis, verification and validation, incorporation of economics and host behaviour.

**Table 3:** Characteristics, construction, parameterization and validation aspects protocol (CCPV protocol) for influenza pandemic model reporting

	**Categories**	**Questions**
General characteristics	Aim of the model	What questions is the model trying to address? Is the model based on past influenza pandemics?
		Is the model aimed at generating predictions for future pandemics used to inform policy making? Are the predictions intended to generate quantitative or qualitative policy insights?
	Theoretical basis	What are the underlying assumptions that support the construction of the model or parts of the model? E.g. the law of mass action, rational choice theory.
	Scale, structure and model type.	What are the geographical and temporal scales of the model? What are the state and control variables and the parameters? Is the model solved analytically through mathematical methods or simulated? What type of model is it?
	Dynamic aspects	Is time modelled as discrete or continuous?
		What variables and processes occur or are updated at each time step?
Construction aspects	Initialization	How is the model initialized? E.g. what proportion of individuals is initially infected?
	Data	Is the model informed by data from previous pandemics? If so, what are the main sources of data in the model?
	Space	Is the model spatially explicit or implicit? What is the spatial structure of the model?
		Are the expected heterogeneities of transmission reflected by this structure?
	Stochasticity	Is the model stochastic or deterministic? How is stochasticity modelled?
	Interventions	What interventions are modelled (e.g. antivirals, vaccination or isolation)? How do the interventions modify epidemiological or clinical parameters in the model?
	Individuals	Are individuals modelled as discrete or continuous entities?
		Are individuals grouped by some characteristic? (e.g. age, risk of infection).
	Interactions leading to transmission	How is interaction between individuals modelled? Are interactions heterogeneous among individuals or locations?
	Economic aspects	Does the model consider the cost of the intervention and/or the economic impact of the disease?
		Does the model seek to guide decision making that will optimise net benefit? Are there groups whose infection would lead to higher economic impacts? Was this distinction considered? Are costs per reduction of disease burden provided?
	Behaviour	Are changes in the behaviour of individuals as a result of pandemic processes being modelled? What are the assumptions made regarding behaviour? Has the model been run without assumptions about pandemic-related changes to behaviour? How do results differ from the model considering such changes?
	Complexity	Have model results been compared with simplified versions of the model? How did results differ?
		To what extent has the increase in complexity in the model hindered its interpretability?
Parameterization and Validation aspects	Sensitivity and uncertainty analysis	Have sensitivity and uncertainty analyses been undertaken? What types of analyses were done, what were the outputs and parameter ranges considered? Were there sensitive or uncertain parameters that were taken directly from previous modelling studies and that might entail a risk of bias to the predictions? Are there alternative data sets to obtain those parameters? Have alternative scenarios for values of those parameters been considered?
	Model parameterization	Describe which parameters were parameterized from: (i) previous parameters used in other pandemic models in the literature; (ii) data published in the literature, e.g. clinical trials, cohort studies; and (iii) pandemic data, e.g. time series of number of cases, attack rates.
		For parameters taken directly from previous pandemic modelling studies, how were these derived? Do they apply to the case being studied? Is there a risk of model overfitting, e.g. by using epidemic case data to fit both transmission and infectious rate parameters?
	Model verification	Has the model undergone standard simulation verification tests? How are results from the model observed to evaluate its functioning? E.g. production of dynamic maps of spread during the simulation.
	Model validation	Has the model been tested for structural and/or predictive validity?
		What type of data independent of model parameterization was used to test its predictive validity? If data were not available for the specific strain of study, did alternative strains or diseases were considered? E.g. seasonal instead of pandemic influenza.
		Was the model able to reproduce the validation data set? If not, what changes to the structure of the model were considered? Did the updated model obtain an improved prediction?
		Was this model developed in parallel with other independent research teams?

## Review of influenza pandemic modelling

**Figure 1:**
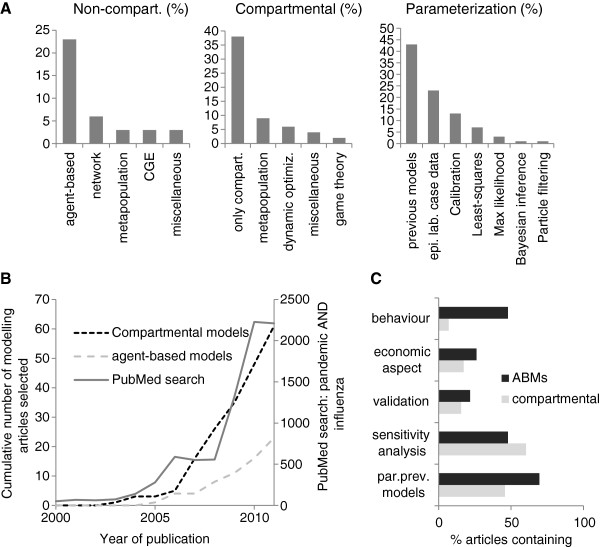
**Literature review of pandemic influenza modelling papers. A**: type of compartmental and non-compartmental models and parameterization approaches used. **B**: cumulative number of modelling and simulation papers identified from 2000 to 2011 (left axis) and number of hits retrieved on PubMed for the query: “pandemic AND influenza” (right axis). This search is used as a surrogate for general research interest in pandemic influenza. **C**: proportion of models incorporating economic aspects, individuals’ behaviour, parameterization from data other than reproducing parameter value choices in previous studies and validation. ABM: agent-based model; CGE: computable or general equilibrium model; Epi. lab. case data: models are fitted to epidemiological, laboratory or case data.

The prevalence of modelling for pandemic influenza has increased dramatically since 2000 (Figure 1B). Out of the 91 articles included in the review, more than half of the models were compartmental (58/91, 64%). Compartmental models were in some instances combined with a dynamic optimization framework (7/58, 12%) or game theory (2/58, 3%). Metapopulation models were employed in combination with both compartmental models and ABMs (9 and 3 models respectively). The second most common modelling approach were ABMs (22/91, 24%). The rest of the models were computable general equilibrium models (CGE) (3/91, 3%), network models (4/91, 4%) and household models (3/91, 3%) (Figure 1A).

Most models that were applied to a specific geographic region focused on high-income countries. Very scarce were studies not focusing on high-income economies (5/91, 6% applied to upper-middle income countries like Thailand or Mexico and none applied exclusively to low-income or lower-middle income countries), despite the higher case fatality rate expected in those countries [32]. The majority of studies were not intended to study impact in specific, localised settings such as schools or hospitals and represented instead the national or international level. A few exceptions did, on the other hand, concentrate on the effects of school closures [4,6,33-37] and on hospitals or hospital staff [38-40].

## Parameterization

In reality many models utilized multiple parameterization strategies. For instance, combining estimates from the literature, censuses and maximum likelihood. For simplicity, models were categorized by the less common and most sophisticated technique used. For instance, a model using literature estimates and Bayesian inference was categorized as using Bayesian methods for parameterization. Among all models, the dominant parameterization strategy (used by 47% of the models) was to adopt parameters from previous studies, especially from other modelling studies perpetuating the use of parameters chosen by other modellers (Figure 1A “parameterization”). 25% of the studies utilized information or parameters derived from epidemiological, laboratory (e.g. viral shedding duration, cohort studies) or case data (e.g. epidemic curves, attack rates) from other sources to parameterize the model. It was common (60%, Figure 1C) to use some sort of sensitivity analysis and this was more frequent in models that did not directly adopt parameters from previous models, suggesting that sensitivity analysis was not used as a complement to reusing parameters from previous models. ABMs were more frequently built using parameters chosen by modellers in previous studies (70% Figure 1C) and constructed from population demographic data, for instance from decennial censuses, rather than using empirical data or parameters obtained from epidemiological or laboratory studies.

Although the most common approach was to parameterise models using parameter values chosen by previous modelling studies, there were several exceptions that used alternative parameterization methods (Figure 1B shows the distribution of parameterization methods and Table 2 defines the methods) ranging from calibration through simulation [33,41,42], maximum likelihood [12,36], least squares [1,11] and Bayesian computational methods such as Markov chain Monte Carlo (MCMC) [4] (Figure 1A).

Several real-time pandemic modelling articles involved sophisticated methods of parameterization employing on-going observed case data, such as maximum likelihood estimation [9] or sequential particle filtering within a Bayesian framework [43]. Their real-time nature enabled the possibility of continuous open validation regarding the prediction of pandemic characteristics such as the timing and height of the peak, and indeed Ong et al. [43] report posting real-time predictions on the internet.

There were several non-real-time examples of modelling papers that parameterized compartmental models using disaggregated epidemic data such as: questionnaire or survey results [44,45]; serological data [36,46]; epidemic cases or mortality time series [1,47-49]; and observed time of pandemic peaks [11,12]. Examples of parameterization from historical epidemic data in ABMs included calibration to reproduce attack or serological infection rates from previous pandemics [33,41,42,50]. Parameterization from case data can be used to investigate policy effectiveness. For instance, Cauchemez et al. [4] evaluated the effectiveness of school closures for pandemic control in France and showed that prolonged school closures would potentially reduce the attack rate of a pandemic by 13–17% by using MCMC Bayesian computational methods to fit an age-structured household-based compartmental model to influenza surveillance data.

Most of the reviewed models reproduced parameter choices from previous studies. This is to be expected as deriving parameters from outbreak data is complex. As a result, some articles specialize in the statistical analysis that leads to parameter derivation and others specialize in the analysis of broad policy questions. There is however the risk that this approach may perpetuate faulty parameterisations from previous studies, or applies a valid parameter value to an inappropriate setting. On the other hand, informing too many parameters in the model by fitting to epidemic time series may run the risk of overfitting or non-identifiability. It may be most credible to inform model parameters using a combination of field or laboratory studies data (e.g. to fit or even directly inform parameters such as recovery rates) and epidemic case data (e.g. to fit transmission related parameters), and then compare fitted parameter values to those obtained from previous studies. One of the possible explanations why this combination of data sources is not common is data paucity, rendering the use of parameters chosen from other modelling studies as one of the few alternatives. One way to increase the pool of available data for model parameterization is to establish international data sharing mechanisms among governments and researchers, especially regarding disease transmission between individuals and surveys of population contact patterns [51], to facilitate the construction of robust models.

Even if epidemic data are available, the small number of models parameterized from such data might also reflect statistical difficulties brought about by censorship in the data—some processes cannot be observed, and many influenza infections are not virologically confirmed, have indistinguishable symptoms, or are asymptomatic. Such censoring combines with non-independence between observations to prevent the use of standard statistical techniques. While such difficulties can be overcome, for instance using maximum likelihood estimation methods [9], particle filtering [43] or other likelihood-based computational methods [4] (Table 3), these require at least some mastery of modern statistical techniques and may be computationally intensive. For instance, Bayesian methods that use MCMC algorithms or approximate Bayesian computation methods can be particularly powerful and flexible tools (Table 2) [52]. These methods allow the merging of prior knowledge on the epidemic parameters—such as those derived from datasets described in the literature—with observed data from the outbreak in question. In addition they allow rigorous parameterisation of models of the processes underlying highly censored data [44]. Bayesian computational methods can thus be used as a flexible and powerful way to perform inference on unobserved parameters. Software such as openBUGS [53] and JAGS [54] are making the use of MCMC algorithms for model fitting accessible to non-specialists.

Parameterization becomes more difficult for large-scale simulation models like ABMs not only because ABMs present many more parameters to be fitted but also because they make it harder to derive an explicit likelihood function making impossible the use of MCMC in Bayesian computational methods or maximum likelihood estimation methods. One promising techniques that does not require full, explicit likelihood functions, and that is used in statistical ecology and DNA sequencing, is one potential solution: sequential importance sampling [55]. Sequential importance sampling, particle filtering or the sequential Monte Carlo method can be performed using the R package POMP [56].

### Implications for CCPV protocol

Reporting the combination of data used for parameterization would allow model users to evaluate the reliability of the models, reduce the risk of model overfitting and allow assessing the adequacy of the parameter for a specific setting (Table 3 “model parameterization”). Sensitivity and uncertainty analysis are other ways to evaluate the influence of individual parameters and their uncertainty range on model predictions (Table 2). They can be used to direct data collection efforts and should ideally be reported (Table 3 “sensitivity and uncertainty analysis”).

## Validation

The review demonstrated the rarity of model validation (only 16% of compartmental models and 22% of ABMs, Figure 1C), despite the importance of two types of validation – structural and predictive (Table 2) – in developing model credibility. Structural validity, which concerns the consistency of a model with theory, may be easier to establish for compartmental models as they are (usually) based on epidemic theory for which results have been derived analytically, as long as they are not oversimplified and unable to capture the salient features of the pandemic. In some instances, modellers may use these analytically soluble models to generate qualitative insights rather than quantitative predictions to inform policy. Structural validity will thus be more relevant for these models rather than comparisons with observed quantitative data.

Predictive validity, on the other hand, is established by comparing model predictions to independently observed outcomes during a pandemic to help assess whether the model appropriately reflects reality, i.e. is capable of capturing the salient mechanisms governing the dynamics of the pandemic. If the agreement with validation data is poor, structural or parametric changes to the model might be needed until adequate validation can be obtained (Table 3). Compartmental models, by aggregating individuals in homogeneous compartments, are amenable to structural changes, accounting, for instance, for spatial and host structure by adding further compartments (e.g. only 64% of the models reviewed were exclusively compartmental with extensions including a metapopulation approach (15%), dynamic optimization (10%) and game theory (3%) (Figure 1A)).

When making structural changes, modellers have to deal with a fundamental trade-off between realism and interpretability, with additional complexity increasing the opacity of the model at the same time it adds realism, potentially up to a point where the model becomes a black box. An example of a structural change is the need to capture spatial hierarchies, such as cities and countries, if space is expected to influence transmission dynamics or the roll out or effectiveness of an intervention. Often such structure is captured using ABMs that represent individuals in different countries, provinces, cities and even districts within a city, but such finely grained structure makes analytical interpretation of model operation virtually impossible. One possible compromise between ease of interpretation and complexity of spatial structure — e.g. between compartmental and ABMs — for populations clustered in cities or countries is the metapopulation model [11,57].

As part of the assessment of predictive validity, it might also be useful to compare models with analogous simplified or extended versions [e.g. [58]. For example, the predictions of a spatially explicit ABM can be compared to those of its “equivalent” spatially implicit compartmental model. Because complex models, such as ABMs, will only be more realistic than compartmental models provided there are data to support their added realism, comparisons of ABMs with their simplified compartmental ‘analogue’ will demonstrate whether the added realism of the ABM is justified by improved predictive power and whether the complexity brought about by the ABM leads to substantial losses in model interpretability (“complexity” in the CCPV protocol, Table 3).

Comparison between models developed by different groups is another interesting alternative to investigate model validity. Parallel model development – by different groups working on the same problem – allows identifying inconsistencies between model results, thus highlighting aspects of the system that are insufficiently understood or outcomes that are not robust to the decisions made in model construction. Parallel model development has been applied for instance to malaria eradication [59], rheumatoid arthritis [60] and HIV antiretroviral treatment effectiveness [61].

If data for validation are non-existent, reporting of the alternative verification techniques used would enhance credibility. These might involve simulation-based observation techniques such as animation (e.g. reproducing maps of model predictions to identify malfunctions), degeneration tests (deactivate model functions to evaluate changes in predictions), extreme-conditions tests (checking that model predictions are logical even under unusually extreme inputs) or face validation (showing results to experts) and can be very useful to detect anomalies in the models [62] (“model verification”, Table 3).

### Implications for CCPV protocol

Reporting the underlying assumptions governing the model, as well as their justification, would help model users evaluate the structural validity of the model (Table 3 “characteristics, theoretical basis”). Validation processes will show if the models are oversimplified and do not capture the salient features of the pandemic. In addition, reporting structural and predictive validity together with subsequent structural changes (e.g. spatial explicitness) to models would allow policy makers to assess the reliability of model predictions, and other analysts to assess the robustness of model construction and parameterisation (Table 3 “construction aspects, space” and “model validation”). Further assistance in evaluating the validity of the model can be obtained through reporting model verification techniques, whether the model has been compared with simpler versions or with other models developed in parallel (Table 3 “model verification” and “complexity”).

## Economic aspects

Very few pandemic preparedness models integrate transmission dynamics and economic analysis [63]. Most models reviewed could quantify the time course of an outbreak and the associated disease and health care endpoints. Metrics such as the reduction in the number infected or dying were commonly used to evaluate the effectiveness of any interventions considered. However, only a minority of studies (17% and 26% of compartmental and ABMs respectively, Figure 1C) sought to address economic questions, either related to the economic impacts of the pandemic or the value for money of the control or mitigation measures in question. In some cases, this may be because epidemiological modellers lack the expertise to identify and model economic aspects. Collaboration between epidemiological modellers and health economists may thus be mutually beneficial to explore new interdisciplinary modelling approaches.

While evaluation of the effectiveness of interventions such as social distancing or antiviral prophylaxis is useful in itself, and may be enough to rule an intervention out or guide policy when costs are uncertain, in many circumstances being able to integrate effectiveness with economic concerns in critical in deciding whether to support the intervention. One possible way to elucidate whether economic aspects would enhance the usefulness of the model for policy makers is to ask whether the relative costs of the intervention would condition its selection. For instance, school closures—identified as effective strategies [4,34,64,65]—of more than four weeks have been shown to burden the economy and even treble the costs arising from an influenza pandemic [66]. In addition, individuals who are economically active will involve a much higher economic burden by job absenteeism due to illness or care giving [67]. Considering the economic impacts of such heterogeneities at a social and individual level may change the optimal implementation of an intervention from what would be recommended based on epidemiological considerations alone (i.e. minimising disease burden). The inclusion of a cost-effectiveness outcome (e.g. cost per quality-adjusted life years (QALY) gained or per case averted) is a common approach which allows comparison of the value for money of different interventions for the same health problem (or even with other health problems when generic measures such as QALYs are used as the denominator).

Few of the reviewed studies incorporated economic aspects but, of those that did, several novel approaches were taken. One such approach was to couple estimates of the cost-effectiveness of vaccinating specific age and risk groups to real-time predictions [9]. These types of real-time outputs of the model, refined as the pandemic progressed, are helpful for decision makers who need to decide the number of vaccine doses to purchase and distribute, and to whom they will be allocated, based on the latest country-specific data.

Novel insights on the optimal allocation of economic resources were also obtained from approaches embedding compartmental models into optimization frameworks such as optimal control theory or dynamic programming [39,45,68-71]. For instance Lee et al. [40], using optimal control theory, identified the optimal way to dynamically allocate control measures such as antiviral allocation and isolation, subject to the dynamics of the pandemic and the effects of the control measures on those dynamics. Their analysis identified aggressive allocation of antivirals at the beginning of the pandemic as an optimal strategy. Accounting for the dynamic nature of the pandemic and allowing control efforts to vary produces new dynamic insights for interventions, a fundamental difference from epidemic models that keep control efforts constant (Table 1).

Few compartmental models were used to perform cost-effectiveness analysis. On those that did, models were integrated in a cost-effectiveness analysis of antiviral prophylaxis and vaccination [72,73]. Cost-effectiveness analyses were also incorporated into ABMs [6,7,74-76]. For instance, Sander et al. [77] estimated the number of QALYs lost and economic costs due to pandemic influenza using a detailed ABM structured by age and infection risk. This model represented people interacting in known contact groups such as households, neighbourhoods, communities, schools and work groups. QALYs were obtained from clinical trial data. Direct costs such as visits to physicians and indirect costs such as job absenteeism were also computed. As a result the cost-effectiveness of different antiviral, school closure and pre-vaccination strategies could be estimated and compared to inform policy making.

The integration of economic and epidemic models for pandemic preparedness does not yet appear to have explored all possible model combinations, with a large scope for modelling innovation. For instance, although advanced economic models such as CGE models have been applied to influenza pandemics and were able to capture the effects of job absenteeism or deaths on the affected sectors of various economies [66,78,79], our review did not identify any study that combined such models with dynamic epidemic models in a way that both models feedback on to each other. Not allowing feedback is reasonable if job absenteeism can be approximated as a sudden shock to the production systems—though in reality the shock might be progressive or present several peaks—or if feedback from the economy into the epidemic is not expected. Examples of such feedback could be changes in individuals’ commuting patterns or behaviour as the economy is affected or a potential loss of the financial capacity to mitigate the epidemic at the individual and government levels.

### Implications for CCPV protocol

Reporting the economic aspects considered in the model, the type of analysis employed, heterogeneity of impacts in different groups and disease burden metrics employed, would facilitate model users understanding the capabilities of the model and the adequacy of the economic analysis undertaken (Table 3, “model construction, economic aspects”).

## Individual behaviour

Behavioural aspects of infection transmission have been studied in the context of the control of other diseases [a general review is provided by [27]. The inclusion of the behaviour of the individuals during an influenza pandemic has heretofore been uncommon among compartmental models and has only recently started to receive attention [10,44,80,81]. Although most pandemic models represent individuals as entities whose behaviour remains invariant, in reality, human behaviour might hinder or foster pandemic mitigation efforts, especially for severe pandemics like that of 1918. Very few compartmental models reviewed considered the effect of changes in behaviour on the impact of the pandemic (7%). New insights have been obtained by integrating compartmental models with game theory [44,80]. For instance, Galvani et al [44] parameterized an epidemiological game-theoretic model from questionnaires on perceptions on influenza. The model was employed to compare self-interested behaviour from the elderly towards vaccination with the socially optimal behaviour that would involve vaccinating children to reduce overall transmission. The model identified how the individual and social equilibria differed more for seasonal influenza than for pandemic influenza – because pandemic influenza might also pose a substantial risk to the young. This study illustrates how, as a result of including human behaviour in the model, the need to incentivize individuals to reduce overall influenza transmission can be identified.

In our review, the inclusion of individuals’ behaviour was more common among simulation models although, instead of basing behaviour representation on game or microeconomic theory, it was usually based on simple rules and assumptions. Different kinds of behaviours were considered in several models, including voluntary isolation, increased social distancing once infected, and preventive behaviour [33,42,66,74,79,82-85]. The inclusion of behaviour can lead to substantially different conclusions. For instance, if individuals perceive an epidemic as life-threatening, they might change their commuting patterns, wear masks and take more extreme precautions [86] and as a result, a model not considering these behavioural changes would overestimate the attack rate and the number of fatalities that eventually would result from the epidemic. In a similar fashion, if individuals perceive an epidemic to be benign, vaccination rates and adoption of precautions may drop, undermining the effectiveness of control measures (evidence of both kinds of responses has been observed during the H1N1 2009 pandemic [81]).

The extent to which human behaviour can affect model predictions is, however, poorly understood and further research is necessary to gauge when behaviour should be included in models. A useful practice would be to report behavioural assumptions, including homogeneity, in the model systematically and how the incorporation of individuals’ behaviour affects model predictions with respect to the model without behaviour (Table 3). Data availability is also a major obstacle for the incorporation of human behaviour to models and again sharing mechanisms would facilitate model development.

### Implications for CCPV protocol

Reporting of the assumptions on how behaviour is modelled would help model users interpreting model results. Reporting of comparisons of model results with and without behaviour would further facility the understanding of behaviour in the model (Table 3, “construction aspects, behaviour”).

## Discussion

Influenza pandemic models have, over the last decade, proliferated dramatically. In parallel to the rapid increase in the number of models, many now incorporate sophisticated parameterization and validation techniques, economic analyses and the behaviour of individuals. Techniques such as Bayesian inference, agent-based modelling and the application of game theory are being newly applied to influenza, answering a more diverse set of public health questions.

This increase in modelling diversity stems from an increase in diversity of research questions and policy strategies. Ultimately, however, the choices made in model construction will depend critically on the data available, the research question and the consideration of the trade-off between realism and interpretability of the model. Even though models need to be fit for purpose, it is noteworthy that many influenza pandemics models rely on parameters from previous modelling studies and are rarely validated using observed data.

Although model validation is not expected in influenza pandemic modelling, it is considered a basic prerequisite for publication in other fields, such as the related discipline of ecological modelling. For instance, the editorial policy of the journal Ecological Modelling states: “Papers that only present a model without support of ecological data for calibration and hopefully also validation of the model will not be accepted because a model has in most cases no interest before it has been held up to ecological reality” [87], and a standardised ODD protocol (overview, design concepts, and details) for documenting ABMs more generally in that field has been published in the same journal [31,88]. Guidelines also exist in the fields of health economics. Examples are guidelines from the National Institute for Clinical Excellence (NICE) in the UK [89], the Drummond Checklist that is required for economic submissions by the British Medical Journal [90], guidelines for cost-effectiveness analysis [91] and modelling guidelines from the International Society for Pharmacoeconomics and Outcomes Research [92].

Given the large variety in modelling approaches for influenza pandemic management and to facilitate comparison between models, we developed a simple general modelling Characteristics, Construction, Parameterization and Validation aspects (CCPV) reporting protocol (Table 3). The use of the protocol together with international data sharing mechanisms would facilitate comparability between models, transparency in decisions about the kinds of models to use, and ultimately increase the confidence in the use of modelling in formulating influenza pandemic policies.

## Competing interests

The authors declare no conflict of interests.

## Authors’ contributions

LRC, ARC made substantial contributions to conception and design. LRC performed data collection and analysis. LRC, ARC, MJ, MIC, VJL analysed and interpreted the data. LRC, ARC, MJ, MIC, VJL and GJM have been involved in drafting the manuscript or revising it critically for important intellectual content. LRC, ARC, MJ, MIC, VJL and GJM have given final approval of the version to be published. All authors read and approved the final manuscript.

## Supplementary Material

Additional file 1: Figure S1 PRISMA 2009 study flow diagram. Click here for file
