# Supplementary material for: Trends in parameterization, economics and host behaviour in influenza pandemic modelling: a review and reporting protocol
Source: Emerg Themes Epidemiol. 2013 May 7;10:3. doi: 10.1186/1742-7622-10-3 (PMC3666982; doi:10.1186/1742-7622-10-3)
Supplement: Additional file 1: Figure S1 — PRISMA 2009 study flow diagram. [file 1742-7622-10-3-S1.doc]

Figure S1. PRISMA 2009 study flow diagram

**Modelling articles and their characteristics.**

Abbreviations: Compart.: compartmental model, metapop.: metapopulation model, ICU: intensive-care unit, ILI: influenza-like illness; ABM: agent-based model; ABM: agent-based model; CGE: computable general equilibrium model.

Table S1. Compartmental-based models 1/5.

| Model type/  description | Case study | Method | Population heterogeneity level | Parameterization | Economic aspects | Behavioral aspects | Validation | Sensitivity analysis | Geographi-cally specific | Source | year |
| --- | --- | --- | --- | --- | --- | --- | --- | --- | --- | --- | --- |
| Compart. | Antivirals and resistance emergence | Simulation | Age-structured | Clinical trials and literature | No | No | No | Yes | Unspecified |  | 2003 |
| Compart. Von Foerster | Identify factors that make an outbreak controllable | Analytical | Homogeneous | From published epidemiological studies. | No | No | No | No | No |  | 2004 |
| Compart. | Estimate R0 of 1918 pandemic. | Simulation | Homogeneous | Minimize sum of squares to fit to death epidemic curves. | No | No | No | Yes | 45 US cities |  | 2004 |
| Compart. Metapop. | Delaying international spread of pandemic influenza | Simulation | Homogeneous by city | Parameterization to historical pandemics. | No | No | No | Yes | Global |  | 2006 |
| Compart. Metapop. | Vaccination, case isolation, antivirals, and air traffic reduction | Simulation | Spatial structure | From previous models | No | No | No | Yes | 52 cities |  | 2006 |
| Compart. Metapop. | H5N1 pandemic international cooperative control | Simulation | Homogeneous within city | Previous models, transport and demographic statistics | No | No | Yes | Yes | Global |  | 2007 |
| Compart. Metapop. | Antivirals and resistance emergence | Analytical | Spatial structure | From previous models | No | No | No | No | Unspecified |  | 2007 |
| Compart. | Evaluation of pandemic strategies effectiveness | Simulation | Age-structure | From previous models | No | Yes | No | Yes | Germany |  | 2007 |
| Compart. Metapop. | Value of international air travel restrictions | Simulation | Homogeneous by city | From previous models | Yes | No | No | Yes | USA |  | 2007 |
| Compart. Game theoretic | Uptake of vaccines from individual and collective interest | Analytical | Age-structure | From surveys and published studies | Yes | Yes | Yes | No | Unspecified |  | 2007 |
| Compart. Metapop. | Antiviral prophylaxis to avoid healthcare workers absenteeism | Simulation | Healthcare workers or not | From previous models | No | No | No | Yes | Singapore |  | 2007 |
| Compart. | Emergence of oseltamivir-resistant strains | Analytical | Homogeneous | From previous models | No | No | No | Yes | Unspecified |  | 2007 |

Table S2. Compartmental-based models 2/5.

| Model type/  description | Case study | Method | Population heterogeneity level | Parameterization | Economic aspects | Behavioral aspects | Validation | Sensitivity analysis | Geographi-cally specific | Source | year |
| --- | --- | --- | --- | --- | --- | --- | --- | --- | --- | --- | --- |
| Compart. | Antiviral contact tracing and prophylaxis versus treatment |  | Homogeneous | From previous models | No | No | No | Yes | Australia |  | 2007 |
| Compart. | Basic control measures, antivirals and vaccines on pandemic influenza control | Simulation | High and low risk classes | From previous models | No | No | No | No | USA, UK, Netherlands |  | 2007 |
| Compart. simulation | Reduction of transmission by closing schools | Simulation | Household | Census data from | No | No | No | Yes | 1 million households, Australia |  | 2007 |
| Compart. Model of immune response | Optimize dose of pre-pandemic vaccination against H5N1 potential pandemic | Simulation | Homogeneous and single high risk group | Infection and field data from past epidemics | No | No | Yes | Yes | Unspecified |  | 2007 |
| Compart.  Integral equation model | Control of influenza pandemic in isolated geographical region |  | Location-structured | From previous models | No | No | No | No | Unspecified |  | 2007 |
| Compart. Metapop. | Effects of internal border control on pandemic influenza spread | Simulation | Homogeneous | From previous models, air travel data | No | No | No | Yes | Cities in Australia |  | 2007 |
| Compart. | AV stockpiling pandemic influenza |  | Homogeneous | From previous models | No | No | No | No | Unspecified |  | 2008 |
| Household compart. | Assess effectiveness of school closure on influenza dynamics | Simulation | Age-structured, household | Bayesian methods MCMC | No | Yes | No | Yes | France |  | 2008 |
| Compart. | Effectiveness of ventilation, wearing masks, vaccination of mitigation in schools | Simulation | Age of the children | Literature and official education statistics | No | No | No | Yes | Taiwan |  | 2008 |
| Compart. | Evaluation of Canada’s pandemic preparedness plan | Simulation | Homogeneous | From previous models and census | No | No | No | No | Canada |  | 2008 |
| Compart. | Effectiveness of antivirals. |  | Homogeneous | From previous models | No | No | No | No | Unspecified |  | 2008 |
| Compart. | Emergence of antiviral resistance | Simulation | Homogeneous | Social studies, viral shedding | No | No | No | Yes | Unspecified |  | 2008 |
| Compart. | Optimal allocation of vaccines using social contact matrix. | Simulation | Age-structure | Social contacts. Observed R0. Literature | No | No | Yes | Yes | Netherlands |  | 2008 |

Table S4. Compartmental-based models 3/5.

| Model type/  description | Case study | Method | Population heterogeneity level | Parameterization | Economic aspects | Behavioral aspects | Validation | Sensitivity analysis | Geographi-cally specific | Source | year |
| --- | --- | --- | --- | --- | --- | --- | --- | --- | --- | --- | --- |
| Compart. | Evaluation of pandemic strategies effectiveness | Simulation | Age and region | From previous models | No | No | No | Yes | Italy |  | 2008 |
| Compart. Stochastic programming. | Optimal vaccination strategies under uncertainty. | Simulation | Homogeneous | Case data, age-contact matrix | Yes | No | No | No | Unspecified |  | 2008 |
| Compart. | Analysis of impact of school closure on 1957 pandemic in the UK | Simulation | Age-structure | Maximum likelihood to consultation, serological and clinical data | No | No | No | Yes | UK |  | 2008 |
| Compart. | AV resistance pandemic influenza | Simulation | Homogeneous | From previous models | No | No | No | Yes | Unspecified |  | 2009 |
| Compart. | Vaccination H1N1 2009 | Simulation | Age-structured | From basic reproduction number and literature | No | No | No | No | Mexico |  | 2009 |
| Compart. Metapop. | Predictions for starting H1N1 2009 pandemic and effect of vaccines | Simulation | Spatial structure | Observed initial parameters | No | No | No | No | 52 cities |  | 2009 |
| Compart. | Control of avian influenza H5N1 potential pandemic | Simulation | Homogeneous | From previous models | No | No | No | No | Unspecified |  | 2009 |
| Compart. Optimal control | Optimal dynamic control of a potential H5N1 pandemic |  | Homogeneous | From previous models | Yes | No | No | No | Unspecified |  | 2009 |
| Compart. Markov model | Cost-effectiveness of vaccination and antivirals stockpiling for potential H5N1 pandemic | Simulation | Homogeneous | From previous models | Yes | No | No | Yes | New York USA |  | 2009 |
| Compart. Optimization | Optimal allocation of vaccines for each group |  | Age-structure | Survey based contact data, literature | Yes | No | No | Yes | USA |  | 2009 |
| Compart. | Vaccination H1N1 2009 | Simulation | Age-structured | From weekly observed data | Yes | No | Yes | Yes | England |  | 2010 |
| Compart. | Effect of antiviral stockpiling and vaccination on H1N1 2009 several waves | Simulation | Homogeneous | From previous models | No | No | No | No | Unspecified |  | 2010 |

Table S3. Compartmental-based models 4/5.

| Model type/  description | Case study | Method | Population heterogeneity level | Parameterization | Economic aspects | Behavioral aspects | Validation | Sensitivity analysis | Geographi-cally specific | Source | year |
| --- | --- | --- | --- | --- | --- | --- | --- | --- | --- | --- | --- |
| Compart. | Vaccination of different age groups. | Simulation | Age-structure | Laboratory studies, transmission matrix. | No | No | No | No | USA |  | 2010 |
| Compart. | Use of oseltamivir and zanamivir to avoid antiviral resistance emergence | Simulation | Homogeneous | From previous models | No | No | No | Yes | Canada |  | 2010 |
| Compart. Optimal control | Effectiveness of antiviral treatment and isolation. |  | Homogeneous | From previous models | Yes | No | No | Yes | Unspecified |  | 2010 |
| Compart. Optimal control | Non-pharmaceutical interventions |  | Homogeneous | From the literature | Yes | No | No | Yes | Unspecified |  | 2010 |
| Compart. optimization | Vaccination of specific age groups in developed and less developed countries at different time points | Simulation | Children-adulsts, low-high risk. | To H1N1 attack rates, literature | No | No | No | Yes | National |  | 2010 |
| Compart. | Antiviral allocation during pandemic | Simulation | Healthcare workers or not | From previous models | No | No | No | Yes | Australia |  | 2010 |
| Compart. | Real-time prediction of H1N1 2009 pandemic | Simulation | Homogeneous | Real time to reported ILIs | No | No | Yes | No | Singapore |  | 2010 |
| Compart. Game theoretic | Uptake of antivirals from individual and collective interest | Simulation | Homogeneous | From census, national-based survey and literature | Yes | Yes | No | No | USA |  | 2010 |
| Compart. | Optimal pandemic influenza vaccine allocation | Simulation | Age-structured and risk | To observed epidemic curve | No | No |  | Yes | Ontario, Canada |  | 2010 |
| Compart. Metapop. | Evaluate value of travel restrictions during the H1N1 2009 pandemic | Simulation | Homogeneous | Maximum likelihood to H1N1 2009 international spread | No | No | Yes | Yes | Global |  | 2011 |
| Compart. | Vaccination |  | Homogeneous | From previous models | No | No | No | Yes | Unspecified |  | 2011 |
| Compart. | Cost-effectiveness of antiviral stockpiling in developed and developing countries | Simulation | Homogeneous | From previous models | Yes | No | No | No | 10 countries |  | 2011 |
| Compart-economic | Effects of adaptive human behaviour on epidemic control predictions | Simulation | Heterogeneous behaviour | Calibrated to reproduce flu-like pathogen | Yes | Yes | No | Yes | Unspecified |  | 2011 |
| Compart. | School closure effect on influenza pandemic, GIS | Simulation | Age-structure, spatial-struct. | Observed pandemic cases and census | No | No | No | Yes | England |  | 2011 |

Table S5. Compartmental-based models 5/5.

| Model type/  description | Case study | Method | Population heterogeneity level | Parameterization | Economic aspects | Behavioral aspects | Validation | Sensitivity analysis | Geographi-cally specific | Source | year |
| --- | --- | --- | --- | --- | --- | --- | --- | --- | --- | --- | --- |
| Compart. With feedback | Resource demand during pandemic in developing countries | Simulation | Clinical severity | From H1N1 2009 pandemic data | Yes | No | No | No | Thailand |  | 2011 |
| Compart. | Optimal antiviral allocation with logistical constraints | Simulation | Homogeneous | Observed ILIs, from literature | No | No | No | Yes | Australia |  | 2011 |
| Compart. | Effect of risk perception on 2009 H1N1 pandemic | Simulation | Normal and altered behaviour | Calibration to ILI incidence data | Yes | Yes | Yes | Yes | Italy |  | 2011 |
| Compart. Optimal control | Control strategies for concurrent seasonal and pandemic influenza |  | Homogeneous | From previous models | Yes | No | No | No | Unspecified |  | 2011 |
| Compart. | Social and biological factors in H1N1 2009 transmission | Simulation | Age-structured | From observed cumulative cases of H1N1 2009. | No | No | No | Yes | Unspecified |  | 2011 |
| Compart. | Antiviral treatment when there is periodic patterns in transmission | Simulation | Homogeneous | From literature and confirmed cases per week H1N1 2009 | No | No | No | No | US |  | 2011 |
| Compart. network | Integrate epidemic dynamics and daily commuting networks | Simulation | Age, spatially-structured | Observed H1N1 2009 cases through time. | No | No | Yes | No | Taiwan |  | 2011 |
| Compart. | Implications of interventions on multiple waves | Simulation | Homogeneous | From previous models | No | No | No | No | Unspecified |  | 2011 |

Table S6. Agent-based models 1/4.

| Model type/  description | Case study | Method | Population heterogeneity level | Parameterization | Economic aspects | Behavioral aspects | Validation | Sensitivity analysis | Geographi-cally specific | Source | year |
| --- | --- | --- | --- | --- | --- | --- | --- | --- | --- | --- | --- |
| ABM | Strategies to contain a potential H5N1 pandemic | Simulation | Individual spatially structured | Census data and kernel fitted to transport. | No | Yes | No | Yes | Thailand, SE Asia |  | 2005 |
| ABM | Strategies to contain a potential influenza pandemic | Simulation | Individual spatially structured | Census data and kernel fitted to transport. | No | Yes | No | Yes | Great Britain, USA |  | 2006 |
| ABM | Mitigation strategies for pandemic influenza H5N1 | Simulation | Individual spatially structured | Census data and to age specific attack rates in past pandemics | No | Yes | No | Yes | USA |  | 2006 |
| ABM | Reduce impact of pandemic with household interventions | Simulation | Household, social network | Household distribution, previous models. | No | No | No | Yes | Honk Kong |  | 2006 |

Table S7. Agent-based models 2/4.

| Model type/  description | Case study | Method | Population heterogeneity level | Parameterization | Economic aspects | Behavioral aspects | Validation | Sensitivity analysis | Geographi-cally specific | Source | year |
| --- | --- | --- | --- | --- | --- | --- | --- | --- | --- | --- | --- |
| ABM | Evaluate scenarios of pandemic control | Simulation | Individual | Census and previous models | No | No | No | No | Italy |  | 2008 |
| ABM | Evaluate effectiveness of targeted containment of influenza pandemic | Simulation | Individual | Age-specific attack rates like 1957 and 1968 pandemics | No | Yes | No | Yes | Chicago, USA |  | 2008 |
| ABM | Effectiveness of school closure on pandemic control | Simulation | Individual | Serologic infection rates. Census and R­0. | No | Yes | No | Yes | Albany, Australia |  | 2008 |
| ABM | Evaluation of effectiveness of pandemic mitigation measures | Simulation | Individual | Census, transport and surveillance data. | No | No | Yes | No | Tokyo, Japan |  | 2008 |
| ABM | Effectiveness of social distancing on pandemic control | Simulation | Individual | Census and previous models | No | No | No | No | Albany, Australia |  | 2009 |
| ABM | Economic evaluation of influenza pandemic mitigation strategies | Simulation | Individual, high-low risk and working or not | Previous models. | Yes | No | No | Yes | Unspecified community, USA |  | 2009 |
| ABM, metapop. | Combination of different antivirals to avoid emergence of resistance | Simulation | Household, social network | Travel and city data, previous models. | No | No | No | Yes | 105 cities |  | 2009 |
| ABM | Epidemic simulation model for influenza control | Simulation | Individual | Calibrated to 195-7-8 and 2009 pandemics | No | No | No | No | USA communities |  | 2010 |
| ABM | Simulation of the protection of healthcare workers | Simulation | Individual | Calibration to the 1957-8 pandemic attack rate. | No | No | No | Yes | Allegheny County, USA |  | 2010 |

Table S8. Agent-based models 3/4.

| Model type/  description | Case study | Method | Population heterogeneity level | Parameterization | Economic aspects | Behavioral aspects | Validation | Sensitivity analysis | Geographi-cally specific | Source | year |
| --- | --- | --- | --- | --- | --- | --- | --- | --- | --- | --- | --- |
| ABM | Vaccine prioritization during H1N1 2009 pandemic | Simulation | Individual, age-structured | Previous models, pandemics, literature, census data. | Yes | No | No | No | Washington DC cities, USA |  | 2010 |
| ABM | Mitigate influenza pandemic with pre-pandemic H5N1 vaccines | Simulation | Individual | Census and previous models. | No | Yes | No | Yes | Albany, Australia |  | 2010 |
| ABM | Evaluate control scenarios against influenza pandemic | Simulation | Individual | Observed previous pandemic, census and transport statistics | No | Yes | No | No | Sapporo, Japan |  | 2010 |
| ABM | Real time prediction of H1N1 2009 pandemic | Simulation | Individual, household | Surveillance data and previous models. | No | No | Yes | No | Italy |  | 2011 |
| ABM | ABM to model pandemic spread with behaviour | Simulation | Individual, household | Census data and previous models | No | Yes | No | No | Ontario, Canada |  | 2011 |
| ABM | Epidemiological and economic effectiveness of reactive control | Simulation | Individual population structured | On illness attack rates and R0. | Yes | Yes | No | No | Hamilton, Canada |  | 2011 |
| ABM network | Economic and social impact of mitigation strategies | Simulation | Individual, income class | Census, income data, previous models. | Yes | Yes | Yes | No | Virginia, USA |  | 2011 |
| ABM | School closure for H1N1 2009 cost-effective | Simulation | Individual | Census data and previous models | Yes | No | No | Yes | Pennsylvania, USA |  | 2011 |
| ABM | Cost-effectiveness of strategies to mitigate pandemic like H1N1 2009 | Simulation | Individual | Census and previous models, H1N1 2009 data | Yes | No | No | No | Albany, Australia |  | 2011 |
| ABM | Effects of word of mouth dynamics of pandemic control | Simulation | Individual | From previous models and observed H1N1 2009 pandemic | No | Yes | No | No | Unspecified |  | 2011 |

Table S9. Miscellaneous models.

| Model type/ description | Case study | Method | Population heterogeneity level | Parameterization | Economic aspects | Behavioral aspects | Validation | Sensitivity analysis | Geographi-cally specific | Source | year |
| --- | --- | --- | --- | --- | --- | --- | --- | --- | --- | --- | --- |
| Network simulation | Effectiveness of targeted antiviral prophylaxis | Simulation | By contact groups and age | previous models | No | No | No | Yes | 2000 persons in the USA |  | 2004 |
| Network  simulation  Genetic algorithms | Optimal vaccination strategies obtained using genetic algorithms | Simulation | Age-structure, community | previous models | No | No | No | No | 10000 individuals |  | 2005 |
| Random graph | Use small-world like network to simulate pandemic interventions | Simulation | Individual | From census, previous models and observed epidemics | No | No | No | Yes | France |  | 2006 |
| Network model | Vaccination H1N1 2009 | Simulation | Age-structured | From previous models and demographic data | No | No | No | No | Vancouver, Canada |  | 2010 |
| CGE | Estimate impacts on the economy of H1N1 pandemic | Simulation | Age-structured, economic sector | Shocks from observed pandemics and previous models | Yes | No | No | No | USA |  | 2010 |
| CGE | Macroeconomic impact of pandemic influenza and different interventions | Simulation | Employed or not | Shocks from epidemic published models and past pandemics | Yes | Yes | No | No | UK, France, Belgium, The Netherlands |  | 2010 |
| Network. Optimization | Estimate optimal geo-temporal distribution of antivirals | Simulation | Homogeneous by city | previous models | No | No | Yes | No | 100 largest cities in USA |  | 2011 |
| Network optimiz. | Dynamic redistribution of scarce pandemic mitigation resources | Simulation | Individual, age, gender, workplace | Historical pandemics, attack rates and R0. | Yes | Yes | No | Yes | Four counties in Florida, USA |  | 2011 |
| CGE | Macroeconomic impact of pandemic influenza and different interventions | Simulation | Age-structured employed or not | Shocks from epidemic published models and past pandemics | Yes | Yes | No | Yes | UK |  | 2011 |

**References**

1. Ferguson N, Mallet S, Jackson H, Roberts N, Ward P: **A population-dynamic model for evaluating the potential spread of drug-resistant influenza virus infections during community-based use of antivirals.** *J Antimicrob Chemother* 2003, **51:**977 - 990.

2. Fraser C, Riley S, Anderson R, Ferguson N: **Factors that make an infectious disease outbreak controllable.** *Proc Natl Acad Sci USA* 2004, **101:**6146 - 6151.

3. Mills C, Robins J, Lipsitch M: **Transmissibility of 1918 pandemic influenza.** *Nature* 2004, **432:**904-906.

4. Cooper B, Pitman R, Edmunds W, Gay N: **Delaying the international spread of a pandemic influenza.** *PLoS Med* 2006, **3:**e212.

5. Flahault A, Vergu E, Coudeville L, Grais R: **Strategies for containing a global influenza pandemic.** *Vaccine* 2006, **24:**6751 - 6755.

6. Colizza V, Barrat A, Barthelemy M, Valleron A, Vespignani A: **Modeling the worldwide spread of pandemic influenza: baseline case and containment interventions.** *PLoS Med* 2007, **4:**e13.

7. Debarre F, Bonhoeffer S, Regoes R: **The effect of population structure on the emergence of drug-resistance during pandemic influenza.** *J R Soc Interface* 2007, **4:**893 - 906.

8. Duerr H, Brockmann S, Piechotowski I, Schwehm M, Eichner M: **Influenza pandemic intervention planning using InfluSim: pharmaceutical and non-pharmaceutical interventions.** *BMC Infect Dis* 2007, **7:**76.

9. Epstein J, Goedecke D, Yu F, Morris R, Wagener D, Bobashev G: **Controlling pandemic flu: the value of international air travel restrictions.** *Plos One* 2007, **2:**e401.

10. Galvani A, Reluga T, Chapman G: **Long-standing influenza vaccination policy is in accord with individual self-interest but not with the utilitarian optimum.** *Proc Natl Acad Sci USA* 2007, **104:**5692 - 5697.

11. Lee VJ, Chen MI: **Effectiveness of neuraminidase inhibitors for preventing staff absenteeism during pandemic influenza.** *Emerging Infectious Diseases* 2007, **13:**449-457.

12. Lipsitch M, Cohen T, Murray M, Levin B: **Antiviral resistance and the control of pandemic influenza.** *PLoS Med* 2007, **4:**e15.

13. McCaw JM, McVernon J: **Prophylaxis or treatment? Optimal use of an antiviral stockpile during an influenza pandemic.** *Mathematical Biosciences* 2007, **209:**336-360.

14. Nuño M, Chowell G, Gumel AB: **Assessing the role of basic control measures, antivirals and vaccine in curtailing pandemic influenza: scenarios for the US, UK and the Netherlands.** *Journal of the Royal Society Interface* 2007, **4:**505-521.

15. Glass K, Barnes B: **How much would closing schools reduce transmission during an influenza pandemic?** *Epidemiology* 2007, **18:**623 - 628.

16. Riley S, Wu J, Leung G: **Optimizing the dose of pre-pandemic influenza vaccines to reduce the infection attack rate.** *PLoS Med* 2007, **4:**e218.

17. Roberts M, Baker M, Jennings L, Sertsou G, Wilson N: **A model for the spread and control of pandemic influenza in an isolated geographical region.** *J R Soc Interface* 2007, **4:**325 - 330.

18. Wood J, Zamani N, MacIntyre C, Beckert N: **Effects of internal border control on spread of pandemic influenza.** *Emerg Infect Dis* 2007, **13:**1038 - 1045.

19. Arinaminpathy N, McLean A: **Antiviral treatment for the control of pandemic influenza: some logistical constraints.** *J R Soc Interface* 2008, **5:**545 - 553.

20. Cauchemez S, Valleron A, Boelle P, Flahault A, Ferguson N: **Estimating the impact of school closure on influenza transmission from Sentinel data.** *Nature* 2008, **452:**750 - 754.

21. Chen S, Liao C: **Modelling control measures to reduce the impact of pandemic influenza among schoolchildren.** *Epidemiol Infect* 2008, **136:**1035 - 1045.

22. Gumel AB, Nuño M, Chowell G: **Mathematical assessment of Canada’s pandemic influenza preparedness plan.** *Can J Infect Dis Med Microbiol* 2008, **19:**185-192.

23. Lunelli A, Pugliese A: **Evaluating the effectiveness of antiviral treatment in models for influenza pandemic.** *Mathematical Medicine and Biology* 2008, **25:**359-372.

24. McCaw J, Wood J, McCaw C, McVernon J: **Impact of emerging antiviral drug resistance on influenza containment and spread: influence of subclinical infection and strategic use of a stockpile containing one or two drugs.** *Plos One* 2008, **4:**e2362.

25. Mylius SD, Hagenaars TJ, Lugner AK, Wallinga J: **Optimal allocation of pandemic influenza vaccine depends on age, risk and timing.** *Vaccine* 2008, **26:**3742-3749.

26. Rizzo C, Lunelli A, Pugliese A, Bella A, Manfredi P, Tomba G, Iannelli M, Degli Atti M: **Scenarios of diffusion and control of an influenza pandemic in Italy.** *Epidemiol Infect* 2008, **136:**1650 - 1657.

27. Tanner MW, Sattenspiel L, Ntaimo L: **Finding optimal vaccination strategies under parameter uncertainty using stochastic programming.** *Mathematical Biosciences* 2008, **215:**144-151.

28. Vynnycky E, Edmunds W: **Analyses of the 1957 (Asian) influenza pandemic in the United Kingdom and the impact of school closures.** *Epidemiol Infect* 2008, **136:**166 - 179.

29. Arino J, Bowman C, Moghadas S: **Antiviral resistance during pandemic influenza: implications for stockpiling and drug use.** *BMC Infectious Diseases* 2009, **9:**8.

30. Chowell G, Viboud C, Wang XH, Bertozzi SM, Miller MA: **Adaptive Vaccination Strategies to Mitigate Pandemic Influenza: Mexico as a Case Study.** *Plos One* 2009, **4**.

31. Flahault A, Vergu E, Boelle P-Y: **Potential for a global dynamic of influenza A (H1N1).** *BMC Infect Dis* 2009, **9:**129.

32. Iwami S, Takeuchi Y, Liu XN: **Avian flu pandemic: Can we prevent it?** *Journal of Theoretical Biology* 2009, **257:**181-190.

33. Jung E, Iwami S, Takeuchi Y, Jo T-C: **Optimal control strategy for prevention of avian influenza pandemic.** *Journal of Theoretical Biology* 2009, **260:**220-229.

34. Khazeni N, Hutton DW, Garber AM, Owens DK: **Effectiveness and cost-effectiveness of expanded antiviral prophylaxis and adjuvanted vaccination strategies for an influenza A (H5N1) pandemic.** *Annals of Internal Medicine* 2009, **151:**840-853.

35. Medlock J, Galvani A: **Optimizing influenza vaccine distribution.** *Science* 2009, **325:**1705 - 1708.

36. Baguelin M, Hoek AJV, Jit M, Flasche S, White PJ, Edmunds WJ: **Vaccination against pandemic influenza A/H1N1v in England: A real-time economic evaluation.** *Vaccine* 2010, **28:**2370-2384.

37. Ghosh S, Heffernan J: **Influenza Pandemic Waves under Various Mitigation Strategies with 2009 H1N1 as a Case Study.** *Plos One* 2010, **5:**e14307.

38. Glasser J, Taneri D, Feng Z, Chuang J-H, Tüll P, Thompson W, Mason McCauley M, Alexander J: **Evaluation of Targeted Influenza Vaccination Strategies via Population Modeling.** *Plos One* 2010, **5:**e12777.

39. Hansen E, Day T, Arino J, Wu J, Moghadas SM: **Strategies for the use of oseltamivir and zanamivir during pandemic outbreaks.** *Can J Infect Dis Med Microbiol* 2010, **21:**e28-63.

40. Lee S, Chowell G, Castillo-Chávez C: **Optimal control for pandemic influenza: The role of limited antiviral treatment and isolation.** *Journal of Theoretical Biology* 2010, **265:**136-150.

41. Lin F, Muthuraman K, Lawley M: **An optimal control theory approach to non-pharmaceutical interventions.** *BMC Infectious Diseases* 2010, **10:**32.

42. Matrajt L, Longini IM, Jr.: **Optimizing vaccine allocation at different points in time during an epidemic.** *Plos One* 2010, **5:**e13767.

43. McVernon J, McCaw JM, Nolan TM: **Modelling strategic use of the national antiviral stockpile during the CONTAIN and SUSTAIN phases of an Australian pandemic influenza response.** *Australian and New Zealand Journal of Public Health* 2010, **34:**113-119.

44. Ong JBS, Chen MI, Cook AR, Lee HC, Lee VJ, Lin RTP, Tambyah PA, Goh LG: **Real-time epidemic monitoring and forecasting of H1N1-2009 using influenza-like illness from general practice and family doctor clinics in Singapore.** *PLoS One* 2010, **5:**e10036.

45. Shim E, Chapman GB, Galvani AP: **Decision Making with Regard to Antiviral Intervention during an Influenza Pandemic.** *Medical Decision Making* 2010, **30:**E64-E81.

46. Tuite AR, Fisman DN, Kwong JC, Greer AL: **Optimal pandemic influenza vaccine allocation strategies for the Canadian population.** *Plos One* 2010, **5:**e10520.

47. Bajardi P, Poletto C, Ramasco JJ, Tizzoni M, Colizza V, Vespignani A: **Human mobility networks, travel restrictions, and the global spread of 2009 H1N1 pandemic.** *Plos One* 2011, **6:**e16591.

48. Bowman C, Arino J, Moghadas S: **Evaluation of vaccination strategies during pandemic outbreaks.** *Mathematical Biosciences and Engineering* 2011, **8:**113 - 122.

49. Carrasco LR, Lee VJ, Chen MI, Matchar DB, Thompson JP, Cook AR: **Strategies for antiviral stockpiling for future influenza pandemics: a global epidemic-economic perspective.** *Journal of the Royal Society Interface* 2011, **8:**1307–1313.

50. Fenichel EP, Castillo-Chavez C, Ceddia MG, Chowell G, Parra PAG, Hickling GJ, Holloway G, Horan R, Morin B, Perrings C, et al: **Adaptive human behavior in epidemiological models.** *Proceedings of the National Academy of Sciences of the United States of America* 2011, **108:**6306-6311.

51. House T, Baguelin M, Van Hoek AJ, White PJ, Sadique Z, Eames K, Read JM, Hens N, Melegaro A, Edmunds WJ, Keeling MJ: **Modelling the impact of local reactive school closures on critical care provision during an influenza pandemic.** *Proceedings of the Royal Society B: Biological Sciences* 2011, **278:**2753-2760.

52. Krumkamp R, Kretzschmar M, Rudge JW, Ahmad A, Hanvoravongchai P, Westenhoefer J, Stein M, Putthasri W, Coker R: **Health service resource needs for pandemic influenza in developing countries: a linked transmission dynamics, interventions and resource demand model.** *Epidemiol Infect* 2011, **139:**59-67.

53. Moss R, McCaw JM, McVernon J: **Diagnosis and Antiviral Intervention Strategies for Mitigating an Influenza Epidemic.** *Plos One* 2011, **6:**e14505.

54. Poletti P, Ajelli M, Merler S: **The effect of risk perception on the 2009 H1N1 pandemic influenza dynamics.** *Plos One* 2011, **6**.

55. Prosper O, Saucedo O, Thompson D, Torres-Garcia G, Wang XH, Castillo-Chavez C: **Modeling control strategies for concurrent epidemics of seasonal and pandemic H1N1 influenza.** *Mathematical Biosciences and Engineering* 2011, **8:**141-170.

56. Simon C, Yosinao N: **A mathematical model to distinguish sociological and biological susceptibility factors in disease transmission in the context of H1N1/09 influenza.** *Journal of Theoretical Biology* 2011, **286:**50-56.

57. Towers S, Vogt Geisse K, Zheng Y, Feng Z: **Antiviral treatment for pandemic influenza: Assessing potential repercussions using a seasonally forced SIR model.** *Journal of Theoretical Biology*.

58. Tsai Y-S, Huang C-Y, Wen T-H, Sun C-T, Yen M-Y: **Integrating epidemic dynamics with daily commuting networks: building a multilayer framework to assess influenza A (H1N1) intervention policies.** *SIMULATION* 2011, **87:**385-405.

59. Wessel L, Hua Y, Wu J, Moghadas S: **Public health interventions for epidemics: implications for multiple infection waves.** *BMC Public Health* 2011, **11:**S2.

60. Ferguson N, Cummings D, Cauchemez S, Fraser C, Riley S, Aronrag M, Iamsirithaworn S, Burke D: **Strategies for containing an emerging influenza pandemic in Southeast Asia.** *Nature* 2005, **437:**209 - 214.

61. Ferguson N, Cummings D, Fraser C, Cajka J, Cooley P, Burke D: **Strategies for mitigating an influenza pandemic.** *Nature* 2006, **442:**448 - 452.

62. Germann T, Kadau K, Longini IMJ, Macken C: **Mitigation strategies for pandemic influenza in the United States.** *Proceedings of the National Academy of Sciences of the United States of America* 2006, **103:**5935 - 5941.

63. Wu J, Riley S, Fraser C, Leung G: **Reducing the impact of the next influenza pandemic using household-based public health interventions.** *PLoS Med* 2006, **3:**e361.

64. Ciofi degli Atti M, Merler S, Rizzo C, Ajelli M, Massari M, Manfredi P, Furlanello C, Scalia Tomba G, Iannelli M: **Mitigation measures for pandemic influenza in Italy: an individual based model considering different scenarios.** *Plos One* 2008, **3:**e1790.

65. Halloran M, Ferguson N, Eubank S, Longini I, Cummings D, Lewis B, Xu S, Fraser C, Vullikanti A, Germann T, et al: **Modeling targeted layered containment of an influenza pandemic in the United States.** *Proc Natl Acad Sci USA* 2008, **105:**4639 - 4644.

66. Milne G, Kelso J, Kelly H, Huband S, McVernon J: **A small community model for the transmission of infectious diseases: comparison of school closure as an intervention in individual-based models of an influenza pandemic.** *Plos One* 2008, **3:**e4005.

67. Yasuda H, Yoshizawa N, Kimura M, Shigematsu M, Matsumoto M, Kawachi S, Oshima M, Yamamoto K, Suzuki K: **Preparedness for the spread of influenza: Prohibition of traffic, school closure, and vaccination of children in the commuter towns of Tokyo.** *Journal of Urban Health-Bulletin of the New York Academy of Medicine* 2008, **85:**619-635.

68. Kelso J, Milne G, Kelly H: **Simulation suggests that rapid activation of social distancing can arrest epidemic development due to a novel strain of influenza.** *BMC Public Health* 2009, **9:**117.

69. Sander B, Nizam A, Garrison LP, Postma MJ, Halloran ME, Longini IM: **Economic Evaluation of Influenza Pandemic Mitigation Strategies in the United States Using a Stochastic Microsimulation Transmission Model.** *Value in Health* 2009, **12:**226-233.

70. Wu J, Leung G, Lipsitch M, Cooper B, Riley S: **Hedging against antiviral resistance during the next influenza pandemic using small stockpiles of an alternative chemotherapy.** *Plos Medicine* 2009, **19:**e1000085.

71. Chao DL, Halloran ME, Obenchain VJ, Longini IM, Jr.: **FluTE, a Publicly Available Stochastic Influenza Epidemic Simulation Model.** *PLoS Comput Biol* 2010, **6:**e1000656.

72. Cooley P, Lee BY, Brown S, Cajka J, Chasteen B, Ganapathi L, Stark JH, Wheaton WD, Wagener DK, Burke DS: **Protecting health care workers: a pandemic simulation based on Allegheny County.** *Influenza and Other Respiratory Viruses* 2010, **4:**61-72.

73. Lee BY, Brown ST, Korch GW, Cooley PC, Zimmerman RK, Wheaton WD, Zimmer SM, Grefenstette JJ, Bailey RR, Assi T-M, Burke DS: **A computer simulation of vaccine prioritization, allocation, and rationing during the 2009 H1N1 influenza pandemic.** *Vaccine* 2010, **28:**4875-4879.

74. Milne G, Kelso J, Kelly H: **Strategies for mitigating an influenza pandemic with pre-pandemic H5N1 vaccines.** *Journal of the Royal Society Interface* 2010, **7:**573-586.

75. Morimoto T, Ishikawa H: **Assessment of intervention strategies against a novel influenza epidemic using an individual-based model.** *Environmental Health and Preventive Medicine* 2010, **15:**151-161.

76. Ajelli M, Merler S, Pugliese A, Rizzo C: **Model predictions and evaluation of possible control strategies for the 2009 A H1N1v influenza pandemic in Italy.** *Epidemiology and Infection* 2011, **139:**68-79.

77. Aleman DM, Wibisono TG, Schwartz B: **A nonhomogeneous agent-based simulation approach to modeling the spread of disease in a pandemic outbreak.** *Interfaces* 2011, **41:**301-315.

78. Andradottir S, Chiu W, Goldsman D, Lee M, Tsui K-L, Sander B, Fisman D, Nizam A: **Reactive strategies for containing developing outbreaks of pandemic influenza.** *BMC Public Health* 2011, **11:**S1.

79. Barrett C, Bisset K, Leidig J, Marathe A, Marathe M: **Economic and social impact of influenza mitigation strategies by demographic class.** *Epidemics* 2011, **3:**19-31.

80. Brown ST, Tai JHY, Bailey RR, Cooley PC, Wheaton WD, Potter MA, Voorhees RE, LeJeune M, Grefenstette JJ, Burke DS, et al: **Would school closure for the 2009 H1N1 influenza epidemic have been worth the cost?: a computational simulation of Pennsylvania.** *BMC Public Health* 2011, **11**.

81. Halder N, Kelso JK, Milne GJ: **Cost-effective strategies for mitigating a future influenza pandemic with H1N1 2009 characteristics.** *Plos One* 2011, **6:**e22087.

82. Loganathan P, Sundaramoorthy S, Lakshminarayanan S: **Modeling information feedback during H1N1 outbreak using stochastic agent-based models.** *Asia-Pacific Journal of Chemical Engineering* 2011, **6:**391-397.

83. Longini I, Halloran M, Nizam A, Yang Y: **Containing pandemic influenza with antiviral agents.** *American Journal of Epidemiology* 2004, **159:**623 - 633.

84. Patel R, Longini IM, Halloran ME: **Finding optimal vaccination strategies for pandemic influenza using genetic algorithms.** *Journal of Theoretical Biology* 2005, **234:**201-212.

85. Carrat F, Luong J, Lao H, Salle A, Lajaunie C, Wackernagel H: **A 'small-world-like' model for comparing interventions aimed at preventing and controlling influenza pandemics.** *BMC Med* 2006, **4:**26.

86. Bansal S, Pourbohloul B, Hupert N, Grenfell B, Meyers LA: **The Shifting Demographic Landscape of Pandemic Influenza.** *Plos One* 2010, **5:**e9360.

87. Dixon PB, Lee B, Muehlenbeck T, Rimmer MT, Rose A, Verikios G: **Effects on the U.S. of an H1N1 epidemic: analysis with a quarterly CGE model.** *Journal of Homeland Security and Emergency Management* 2010, **7:**article75.

88. Keogh-Brown MR, Smith RD, Edmunds JW, Beutels P: **The macroeconomic impact of pandemic influenza: estimates from models of the United Kingdom, France, Belgium and The Netherlands.** *European Journal of Health Economics* 2010, **11:**543–554.

89. Dimitrov NB, Goll S, Hupert N, Pourbohloul B, Meyers LA: **Optimizing Tactics for Use of the U.S. Antiviral Strategic National Stockpile for Pandemic Influenza.** *Plos One* 2011, **6:**e16094.

90. Savachkin A, Uribe A: **Dynamic redistribution of mitigation resources during influenza pandemics.** *Socio-Economic Planning Sciences* In Press.

91. Smith RD, Keogh-Brown MR, Barnett T: **Estimating the economic impact of pandemic influenza: An application of the computable general equilibrium model to the UK.** *Social Science & Medicine* 2011, **73:**235-244.
